# Effect of Oral Antimicrobial Peptide R7I Against Epidemic Enteropathogenic *Neisseria* in Geese (*Anser cygnoides orientalis*)

**DOI:** 10.3390/ani15202939

**Published:** 2025-10-10

**Authors:** Shuo Jia, Junhan Gao, Jing Fu, Chongpeng Bi, Xiujing Dou, Anshan Shan

**Affiliations:** 1 College of Animal Science and Technology, Northeast Agricultural University, Harbin 150038, China; jiashuo@neau.edu.cn (S.J.); gjh7573@163.com (J.G.); bnm0722@163.com (C.B.); 2 College of Veterinary Medicine, Northeast Agricultural University, Harbin 150038, China; 3 College of Animal Science and Technology, Zhongkai University of Agriculture and Engineering, Guangzhou 510408, China; fujing@neau.edu.cn

**Keywords:** antimicrobial peptides, *Neisseria*, gut microbiota, inflammation, PPAR signaling pathway

## Abstract

**Simple Summary:**

The emergence of antibiotic-resistance bacteria causes the lack of available drugs used in disease treatments. Antimicrobial peptides (AMPs) are green and safe antibiotic alternatives. In 2022, an outbreak of an acute diarrheal disease caused by Gram-negative cocci named *Neisseria* occurred among domestic geese (*Anser cygnoides orientalis*) in Northeast China. Here, we analyzed *Neisseria* S1, which caused diarrhea in goose, and determined whether AMPs R7I designed in our laboratory can be used in *Neisseria* infection. This is the first report and isolation of enteropathogenic *Neisseria*, as well as the first report of *Neisseria* infection in *Anser cygnoides orientalis*. Our results showed that peptide R7I has the capability to counteract *Neisseria* S1 infection both in vivo and in vitro. R7I can be used as an oral antibiotic alternative in the feed.

**Abstract:**

The emergence of antibiotic-resistant bacteria has resulted in a lack of available drugs that can be used to treat various diseases. Antimicrobial peptides (AMPs) are green and safe antibiotic alternatives. In 2022, an outbreak of an acute diarrheal disease caused by Gram-negative cocci named *Neisseria* occurred among domestic geese (*Anser cygnoides orientalis*) in Northeast China. In this study, we analyzed *Neisseria* S1, which caused diarrhea in geese, and determined whether AMP R7I designed in our laboratory can be used to treat *Neisseria* infection. This is the first report and isolation of enteropathogenic *Neisseria*, as well as the first report of *Neisseria* infection in *Anser cygnoides orientalis*. Peptide R7I has the capability to counteract *Neisseria* S1 infection both in vivo and in vitro. R7I induced the release of intracellular contents, leading to the death of *Neisseria*. Oral treatment of R7I modulated metabolic levels, antioxidant capacity, and immune responses and inhibited inflammation in *Neisseria*-infected geese. Furthermore, R7I significantly contributed to the recovery of intestinal homeostasis and regulated intestinal function via a signaling pathway related to metabolism in *Neisseria* infection. During our study of the mechanism of R7I against *Neisseria* infection, we preliminary found that R7I regulates lipid metabolism disorder and inflammation caused by *Neisseria* infection through the PPAR signaling pathway. In conclusion, R7I shows a strong ability against *Neisseria* infection, and it can be used as an oral antibiotic alternative in animal feed.

## 1. Introduction

With the emergence of antibiotic-resistant bacteria, there are fewer antibiotics available for clinical use. Antibacterial peptides (AMPs) are widely considered broad-spectrum natural antibiotics with pathogen-killing/inhibiting capabilities. Currently, AMPs are extensively investigated as potential substitutes for antibiotics in combating bacterial infections [1,2,3]. However, current AMP drugs approved by the Food and Drug Administration (FDA) face certain difficulties in oral administration due to susceptibility to proteolytic enzyme digestion (such as Daptomycin produced by *Streptomyces roseosporus*, Polymyxin B produced by *Bacillius polymyxa*), yet oral administration is the recommended technique for treating gastrointestinal inflammatory illnesses [1,4,5]. AMP R7I designed in our lab has good anti-enzymatic stability, broad-spectrum antibacterial ability, and the capacity to protect the intestinal barrier from infection with intestinal pathogens while also inhibiting the proliferation of intestinal pathogens. R7I overcomes the shortcomings of traditional AMPs and has become a potential new oral antimicrobial agent for treating antibiotic-resistant bacterial infections. Although it has been confirmed that R7I has antibacterial ability against Gram-negative bacilli (*Escherichia coli* and *Salmonella typhimurium*) in mice, its effectiveness against Gram-negative cocci in other animal models remains unverified [1,2,6].

The genus *Neisseria*, a group of Gram-negative diplococcus, colonizes on the surface of the mouth, oropharynx, and genital tract mucosa and is typically considered a normal microbiota in healthy hosts [7,8]. However, due to various reasons (such as the evolution of various species, changes in colonizes locations, and immune system damage), several strains have been considered pathogenic for humans and animals, especially in hosts with impaired immune function [9,10,11,12,13,14,15,16]. Beyond urogenital and oropharyngeal infections, *Neisseria* can also lead to diarrhea and neurological deficits. Except urogenital and oropharyngeal infections, *Neisseria* can also lead to diarrhea and neurological deficit [16,17,18]. According to WHO estimates, there are over 106 million human cases worldwide annually. Furthermore, the positive rates of *Neisseria* in humans across several countries can reach 30% [19,20]. In addition to humans, *Neisseria* is also widespread in animals. The positive rate of *Neisseria* was 80–88% in 1-month-old sheep and 20.5–79.3% in old sheep in the 1990s in Israel [21]. In the poultry industry, in 2015, researchers found that in the Arctic, the positive rate of *Neisseria* in wild geese (*Anser anser*) was 66.7%, while the lethal rate in white-fronted geese (*Anser albifrons*) was more than 70% [7,22]. In addition, the infection rate of *Neisseria* in Chinese ducks was 8% and the mortality rate was 20% in 2014 [14]. Although there was no clear epidemiological investigation, in 1973, researchers in New York found that the infection rate of *Neisseria* in chicken embryos was 12–69%, demonstrating that *Neisseria* is also a threat to chickens [23,24].

Research shows that *Neisseria* has evolved antibiotic resistance mechanisms [18], with numerous clinical strains demonstrating resistance to most available antibiotics (including sulfonamides, penicillin, quinolones, and cephalosporins), which highlights the urgent need for novel antimicrobials targeting *Neisseria* infection [19,25]. Natural AMPs including LL-37, LSA-5, Bmkn2, TP-1, HE2, PG-1, and Mutacin B-Ny266 have demonstrated anti-*Neisseria* activity via outer membrane disruption [26]. However, the existence of an energy-dependent efflux system endows *Neisseria* with resistance to natural AMPs, decreasing their effect [27]. Furthermore, the lack of in vivo studies, especially regarding AMPs’ anti-*Neisseria* mechanism and immune methods in the host, restricts the application of anti-*Neisseria* peptides [25,28,29].

To develop novel anti-Gram-negative cocci agents, we used *Neisseria* S1 (isolated from the intestine of diarrheal geese) as a bacteria model and evaluated the anti-*Neisseria* activity of AMP R7I (designed in our lab and exhibiting great antibacterial activity against various bacterial infections) both in vivo and in vitro. The findings of this study will highlight the therapeutic potential of R7I in treating Gram-negative cocci infection and have the potential to provide a theoretical basis for the further application of anti-enzymatic peptide R7I as an oral antibiotic alternative in animal feed.

## 2. Materials and Methods

### 2.1. Animal Ethics Statement

This study involves animal subjects and was approved by the Northeast Agricultural University Institutional Animal Care and Use Committee (approval no. NEAUEC20230695) on 10 September 2023.

### 2.2. Design and Synthesis of R7I

AMP R7I (IRPIIRPIIRPIIRPIIRPIIRPIIRPI-NH2) with good anti-enzymatic stability and broad-spectrum antibacterial ability was designed in our laboratory. It has been demonstrated that R7I has low toxicity and the ability to defend against intestinal pathogen infection in vitro and in mice [1,2,6]. R7I was synthesized by GL Biochem Corporation (Shanghai, China) and determined by matrix-assisted laser desorption/ionization time-of-flight mass spectrometry (MALDI-TOF MS; Linear Scientific Inc., Milpitas, CA, USA). Peptide purity (>95%) and retention time were tested using reverse-phase high-performance liquid chromatography (HPLC) with a GS-120-5-C18-BIO 4.6 × 250 mm^2^, 220 nm column, 10 μL in volume, using a nonlinear water/acetonitrile gradient that contained 0.1% trifluoroacetic acid at a flow rate of 1.0 mL/min [6].

### 2.3. Disease Outbreak and Sample Collection

In June 2022, an acute diarrheal disease outbreak occurred among 5–15-day-old domestic geese across farms of the Heilongjiang Province. On these farms, there were vaccination records for the last 3 years regarding vaccines against Avian cholera, Newcastle disease virus, Avian influenza virus, and Goose Parvovirus. The main clinical symptoms were diarrhea, enteritis, and secondary convulsion death. On the infected farm, the infection rate was 82.9% and the mortality rate was 50.3%. The liver, lungs, and intestine of the dead geese were collected for further analysis.

### 2.4. Isolation and Biological Characteristics of Neisseria

The liver, lung, and intestine tissues were homogenized in aseptic mortar with sterile Phosphate-Buffered Saline (PBS, Beyotime, Shanghai, China), followed by plate cultivation (plate streaking method using sterile contact ring) on bouillon culture medium and incubation for 12 h. Single colonies were cultured on Tryptic Soy Broth (TSB, Hopebio, Qingdao, China) agar plates at 37 °C for 12 h, and then isolated and cultured in TSB at 37 °C and 200 rpm for 12 h. Gram’s staining (using commercial kits by Solarbio, Beijing, China), scanning electron microscopy (SEM, Hitachi, Tokyo, Japan), and transmission electron microscopy (TEM, Hitachi, Japan) were employed for morphological observation. For SEM observation, the bacteria were harvested and washed with PBS; 2.5% glutaraldehyde was used for fixation; 50%, 70%, 90%, and 100% ethanol were used for gradient dehydration; and a 1:1 mixture of ethanol and tert butyl alcohol alongside pure tert butyl alcohol were used for replacement. Finally, drying and observation were carried out. For TEM observation, the bacteria were harvested and washed with PBS; 1% osmic acid was used for fixation; 50%, 70%, 90%, and 100% ethanol were used for gradient dehydration; a 1:1 mixture of ethanol and acetone alongside pure acetone were used for replacement; and a 1:1 mixture of acetone and resin and 100% resin were used for embedding. Finally, the observation was carried out.

Total DNA of the bacteria was extracted according to the steps in the commercial kits’ manual (K7022, Thermo Scientific, Waltham, MA, USA), followed by PCR with PCR Master Mix (Beyotime, Beijing, China) and Primers 27F and 1429R (Listed in Table 1). The PCR reaction system is as follows: PCR Mix at 10 μL, DNA at 1 μg, each primer at 1 μM, and ddH_2_O up to 20 μL. The reaction procedure is as follows: STEP1, 94 °C for 3 min; STEP2, 94 °C for 30 s; STEP3, 55 °C for 30 s; STEP4, 72 °C for 1 min; STEP5, repeat STEP2 for 30 cycles; STEP6, 72 °C for 10 min; STEP7, 4 °C for the remaining time. After that, 16S rDNA sequencing was carried out by Sangon Biotech (Shanghai, China). The sequence of the 16S rDNA was aligned using the BLAST tool from NCBI (https://blast.ncbi.nlm.nih.gov/Blast.cgi, accessed on 8 October 2025).

*Neisseria* was cultured in TSB, supplemented with 5% Fetal Bovine Serum (FBS, Gibco, Waltham, MA, USA) at 37 °C for 16 h, and the OD_600_ was determined by using BioSpectrometer basic (Eppendorf, Hamburg, Germany) every 2 h to construct the growth curve of *Neisseria*. Antibiotic susceptibility tests were performed using commercial drug sensitivity test strips (Drug sensitive paper, BKMAM, Changde, China) following the manufacturer’s instructions and judged against Performance Standards for Antimicrobial Susceptibility Testing (M100) [30]. The drug sensitivity test strips included gentamicin (30 μg/piece), neomycin (30 μg/piece), amikacin (30 μg/piece), streptomycin (30 μg/piece), ofloxacin (5 μg/piece), ciprofloxacin (5 μg/piece), enrofloxacin (5 μg/piece), amoxicillin (30 μg/piece), ampicillin (30 μg/piece), cefazolin (30 μg/piece), penicillin (30 μg/piece), erythromycin (10 μg/piece), azithromycin (10 μg/piece), compound sulfamethoxazole tablets (20 μg/piece), doxycycline (30 μg/piece), fosfomycin (10 μg/piece), vancomycin (30 μg/piece), and lincomycin (10 μg/piece).

Phylogenetic analysis was performed based on the 16S rDNA sequence using the Neighbor-Joining method with 1000 bootstrap replicates in Mega 7 software. Complete genome sequencing was performed by Biomarker Biotechnology Co., Ltd., Beijing, China. In brief, the total genome DNA of *Neisseria* was extracted by using commercial kits according to the manufacturer’s instructions (K7022, Thermo Scientific, USA) and then sequenced on the Illumina sequencing platform. The filtered reads were assembled using SPAdes v3.6.2 software. Genome component prediction was performed with Prodigal v2.6.3 software. The Non-redundant (Nr) protein database, Gene ontology (GO) database, Kyoto Encyclopedia of Genes and Genomes (KEGG) database, and Evolutionary Genealogy of Genes: Non-supervised Orthologous Groups (eggNOG) database were used for functional annotation with an e-value threshold of 1 × 10^−5^.

**Table 1:** Primers used in this study.

Target Gene	Primers	Sources
16S	27F: AGAGTTTGATCCTGGCTCAG 1429R: GGTTACCTTGTTACGACTT	Published source [31]
PPARα	F: AGGTGATGATAGCTCTGGAGCT R: TGTTTAATGCTCCACTGGGT	Designed de novo
PPARβ	F: GCCAGTACTGCCGCTTCCAG R: CCTGTGGGTTCTGGCAGCTGATCTC	Designed de novo
PPARγ	F: CACAAGCGGAGAAGGAGAAGCTCC R: AAGATCGCCCTCGCCTTGGC	Designed de novo
FABP1	F: GTTCAAGGTTACCGTCACC R: TCTTGCTGATTCTCTTGTAGGTG	Designed de novo
FABP2	F: GGTGTTAACATTATGAAAAGGAAG R: CAGTAAGTTCAGTCCCATCTG	Designed de novo
ACO	F: GGCCTGTGGTGGGCATGGCTATTC R: GGCTGTCTGCAGCATCATGAC	Designed de novo
SCD-1	F: GGATCGTCATGAGAAGACTTACTC R: TCAGTGTCAACCCGAATATGG	Designed de novo
CD36	F: CCTTACACGTACAGGGTGCG R: CTGTCCCAACAGACATATCAGG	Designed de novo
β-actin	F: CGTGCTGTCGCTGTACGCCTCCG R: GGATGGCATGGGGCAGAGCGTAGCC	Designed de novo

### 2.5. Assessment of Anti-Neisseria Activity of R7I

The antibacterial activity of R7I was measured following a previously described method with modifications [6]. Briefly, the improved broth dilution method is as follows: *Neisseria* S1 in the mid-logarithmic phase (8 h) was diluted with TSB to a final concentration of 1 × 10^5^ CFU/mL (the concentration was detected by using colony plate counting method and OD_600_). A volume of 50 μL of the bacterial suspension was incubated with 50 μL of different concentrations (64–1 μM) of peptides in a sterile transparent 96-well plate (CLS3340, Merck, Darmstadt, Germany). The plate was incubated at 37 °C for 24 h (Heratherm constant-temperature incubator, 51028135, Thermo Fisher Scientific, USA; Reacti-Therm™ thermometer, TS-18914, Thermo Fisher Scientific, USA; Traceable™ Laboratory bench timer, Thermo Fisher Scientific, USA), and the OD_600_ was measured by using an automatic microplate reader (Multiskan™ FC, 1410101, Thermo Fisher Scientific, USA) to calculate the minimum inhibitory concentration (MIC). The MIC was defined as the lowest peptide concentration that inhibited 95% of *Neisseria* S1 growth, expressed in μM. Furthermore, to identify the minimum bactericidal concentration (MBC), the plate was incubated at 37 °C for 4 h, then 50 μL of the mixture was taken out from each well, diluted with 50 μL TSB in a new sterile 96-well plate, and cultured at 37 °C for 16 h, and the OD_600_ was measured to calculate the MBC. The lowest peptide concentration that killed 99.9% of the *Neisseria* S1 was the MBC (μM). The experiment was repeated three times independently, and three parallel tests were conducted each time.

To determine the bactericidal kinetics of R7I against *Neisseria*, *Neisseria* S1 at a cell density of 1 × 10^5^ CFU/mL was co-incubated with R7I at a 1 × MBC. Bacteria survival rates were determined using the plate method at different time points (0, 15, 30, 60, 90, 180, 300, 600, and 900 s) post co-incubation. The experiment was repeated three times independently, and three parallel tests were conducted each time.

### 2.6. Anti-Neisseria Effect of R7I In Vitro

To calculate the influence of R7I on *Neisseria* outer membrane permeabilization, *Neisseria* S1 was cultured to OD_600_ = 0.2 and incubated with 1-N-phenylnaphthylamine (NPN, 10 μM) for 30 min. The suspension was then mixed with equal volumes of different concentrations of R7I (1–64 μM) in a sterile 96-well black plate. Fluorescence was recorded, and outer membrane permeabilization was calculated as previously described [4].

To analyze the permeabilization of the inner membrane, *Neisseria* S1 was cultured to OD_600_ = 1.0, centrifuged and diluted in 5 mM HEPES solution (pH = 7.4, containing 20 mM glucose and 1.5 mM o-nitrophenyl-β-Dgalactoside, ONPG), and diluted to OD_600_ = 0.1. Subsequently, equal volumes of different concentrations of R7I (8–64 μM) and bacterial suspension were added to a sterile 96-well plate and incubated at 37 °C for 1 h. The OD_420_ was measured every 6 min [6].

*Neisseria* S1 was cultured to OD_600_ = 0.2, followed by co-incubation with R7I at 1 × MBC for 1 h, and the changes in bacteria were observed by scanning electron microscopy (SEM) and transmission electron microscopy (TEM).

### 2.7. Concentration of R7I Used In Vivo

A total of 65 one-week-old Sanhua geese were purchased from Muwa Goose Breeding Co., Ltd. (Harbin, China). The geese were randomly divided into two groups: 25 for this section, and 40 for further experiments. In this section, geese were randomly divided into five groups: Mock (geese fed with normal food without any special treatment), 5 mg/kg R7I, 10 mg/kg R7I, 20 mg/kg R7I, and 40 mg/kg R7I (n = 5 per group, different groups were kept in different cages). They were acclimated with food and environmental training for 7 days. The geese in groups receiving different concentrations of R7I were orally administered R7I (5, 10, 20, 40 mg/kg) for 3 days continuously, while the Mock group were orally immunized with equal volumes of sterile PBS. Measurements of the weight of geese in each group were recorded at 14, 21, and 28 days after the geese hatched. On the 7th day post immunization, blood from each group was collected, and serum biochemical indexes including alanine aminotransferase (ALT), which reflects liver function, urea nitrogen (BUN), which reflects renal function, and lactate dehydrogenase (LDH), which reflects inflammatory response, were measured by using an automatic biochemical analyzer (Roche, Basel, Switzerland).

### 2.8. Anti-Neisseria Effect of R7I In Vivo

A total of 40 geese were obtained from Muwa Goose Breeding Co., Ltd. (Harbin, China) and randomly divided into four groups (n = 10 per group, different groups were kept in different cages): Neisseria, R7I, Neomycin (*Neisseria* S1 is sensitive to Neomycin), and Mock (geese fed with normal food without any special infection or treatment). They were acclimated with food and environmental training for 7 days. (In this study, Neisseria is the group name, and *Neisseria* means the isolation strain).

The geese in the Neisseria, R7I, and Neomycin groups were orally infected with 2 × 10^8^ CFU of *Neisseria* S1 for 3 days continuously, while the Mock group were orally immunized with 1 mL sterile PBS. Subsequently, the R7I and Neomycin groups were orally administered R7I (20 mg/kg) or Neomycin (20 mg/kg), respectively, for 3 days continuously, whereas the Neisseria and Mock groups were orally immunized with equal volumes of sterile PBS.

**Figure 1:**
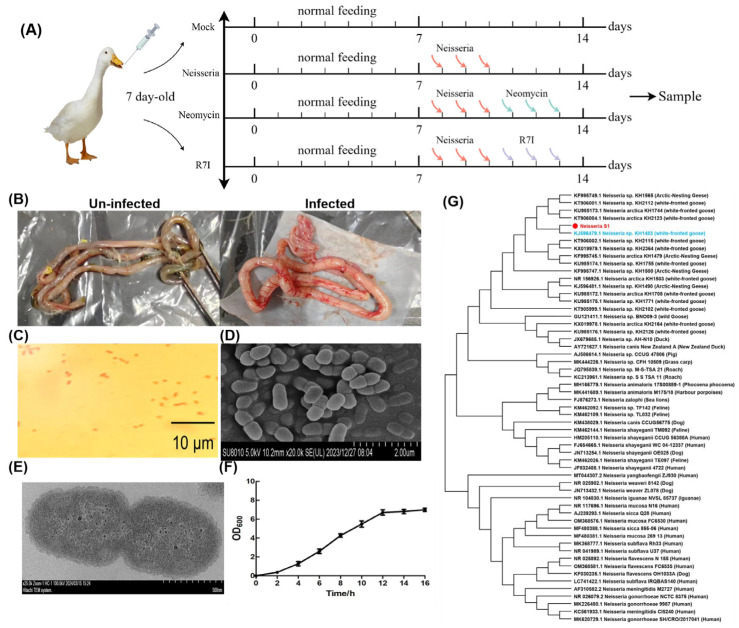
Isolation and characterization of *Neisseria* S1 strain. (**A**) The immune program of this study. (**B**) Pathological results of infected geese. The intestines were swollen in infected geese. (**C**) The Gram staining results of the isolated strain. The isolated strain is a kind of Gram-negative bacteria. (**D**,**E**) Morphological observation of the isolated strain by SEM (**D**) and TEM (**E**). The isolated strain is diplococcus. (**F**) A one-step growth curve. 0–2 h is the lag phase, while 4–12 h is the logarithmic growth phase and 12–16 h is the plateau phase. (**G**) Phylogenetic analysis of 16S rDNA genes of *Neisseria* S1 strain and other strains found in GenBank. *Neisseria* S1 (the red one) has the highest sequence homology to the strain KH1483 (the blue one).

Daily measurements of body weight, weight gain, and survival rates of each group were recorded. On the 7th day post-infection, the intestines, thymus, bursa of Fabricius, and spleen from each group were collected, and histopathological changes were assessed via pathoanatomical analysis and hematoxylin–eosin (HE) staining carried out by the Harbin Veterinary Research Institute. The pathologists were blind to the clinical grouping (e.g., *Neisseria* infection group, or R7I treatment group) during the evaluation process. In addition, pathological evaluation followed the previous grading standards of the Harbin Veterinary Research Institute, including classification criteria for inflammation severity, cell damage, and cell infiltration, and was performed by two professionals who made separate judgments. Blood and intestinal mucosal lavage fluid from each group were collected and kept at −80 °C for further analysis. The infection process is shown in Figure 1A, created using Figdraw (https://www.figdraw.com/).

### 2.9. Measurement of Serum Biochemical Indexes, Antibodies, and Cytokines

To determine functional changes in different tissues in geese, serum biochemical indexes, including alanine aminotransferase (ALT), aspartate aminotransferase (AST), alkaline phosphatase (ALP), glutamyl transferase (GGT), and cholinesterase (CHE), which reflect liver function; urea nitrogen (BUN), creatinine (CREA), and uric acid (UA), which reflect renal function; α-hydroxybutyrate dehydrogenase (HBDH), creatine kinase (CK), and creatine phosphokinase isoenzyme (CKMB), which reflect cardiac function; and lactate dehydrogenase (LDH), which reflects inflammatory response, were measured by using an automatic biochemical analyzer (Roche, Switzerland).

The levels of lipid-metabolism-associated indexes—total cholesterol (TC), triglyceride (TG), high-density lipoprotein cholesterol (HDL-C), and low-density cholesterol (LDL-C)—and antioxidant-function-associated indexes, namely, superoxide dismutase (SOD), catalase (CAT), total antioxidant capacity (T-AOC), glutathione (GSH), and malondialdehyde (MDA), in serum were detected using commercially available kits (Jiancheng, Nanjing, China). The item numbers are as follows: TC, A111-1-1; TG, A110-1-1; HDL-C, A112-1-1; LDL-C, A113-1-1; SOD, A001-3-2; CAT, A007-1-1; T-AOC, A015-2-1; GSH, A005-1-2; MDA, A003-1-2. Detection was performed according to the manufacturer’s instructions and measured by using an automatic microplate reader (Multiskan™ FC, 1410101, Thermo Fisher Scientific, USA).

The levels of IgG antibodies and cytokines (IL-1β, IL-6, IL-8, IL-10, IFN-γ, TNF-α) in serum and sIgA in intestinal mucus, which showed levels of humoral immunity and cellular immunity, were determined using commercial enzyme-linked immunosorbent assay (ELISA) kits (Jingmei, Yancheng, China). The item numbers are as follows: IgG, JM-060779O2; IL-1β, JM-00921C2; IL-6, JM-00102O2; IL-8, JM-00447D2; IL-10, JM-00467D2; IFN-γ, JM-00446D2; TNF-α, JM-00869C2; sIgA, JM-08029D2. Detection was performed according to the manufacturer’s instructions and measured by using an automatic microplate reader (Multiskan™ FC, 1410101, Thermo Fisher Scientific, USA).

The indexes mentioned above in each group were compared with the levels in the Mock group.

### 2.10. Gut Microbiota Analysis

On the 7th day post infection, the cecal contents of each group were scraped and collected and then preserved with dry ice and transported to the laboratory for sequencing. The levels of intestinal microbiota from the cecal contents of each group were measured via 16S rDNA sequencing performed using the Illumina NovaSeq sequencing platform by Shanghai Personal Biotechnology Co., Ltd., Shanghai, China. The results were analyzed as previously described [1]. The DADA2 method under default parameters was used for primer removal, quality filtering, denoising, splicing, and clustering. Greengenes database (Release 13.8, http://greengenes.lbl.gov/) under default parameters was selected for species annotation.

### 2.11. RNA-Seq Analysis

On the 7th day post infection, the intestines of each group were collected, and then preserved with dry ice and transported to the laboratory for sequencing. Sequencing was performed by Shanghai Personal Biotechnology Co., Ltd., Shanghai, China. In brief, the total RNA from the intestines of the Neisseria and R7I groups was extracted by using TRIzol reagent (15596026CN, Thermo Scientific, USA) according to the manufacturer’s instructions. Subsequently, the cDNA libraries were constructed and sequenced using the Illumina NovaSeq platform. The filtered reads were aligned to the goose reference genome (GCF_000971095.1_AnsCyg_PRJNA183603_v1.0_genomic.fna) using HISAT2 software (http://ccb.jhu.edu/software/hisat2/index.shtml). FPKM (Fragments Per Kilo bases per Million fragments) were used for normalization, and genes with FPKM > 1 were considered to be expressed. DESeq was used to analyze gene expression differences, and the genes with a |log2FoldChange| > 1 and *p*-value < 0.05 were identified as significantly differentially expressed genes (DEGs). Gene ontology (GO) database analysis (*p*-value < 0.05) was used for differential gene functional annotation, while the Kyoto Encyclopedia of Genes and Genomes (KEGG) database analysis (*p*-value < 0.05) was used to identify the differential enrichment pathways.

### 2.12. Regulation of Lipid Metabolism via PPAR Pathway

The intestinal epithelial cells of geese (IECs) were isolated from 25 embryo-age geese eggs (obtained from Muwa Goose Breeding Co., Ltd., Harbin, China) and kept in our laboratory. In brief, the intestine tissues of geese eggs were collected under sterile conditions, washed with sterile PBS three times, then cut with sterile and transferred to Collagenase I solution (1 mg/mL, Solarbio, China) for digestion. After 3 h, we added RPMI-1640 medium (Gibco, USA) containing 10% FBS (Gibco, USA) to stop digestion, and used a gauze for filtering, collecting sterile cells, and transferring the filtered culture medium to a new sterile cell bottle for culture. The culture was frozen and stored for use until the cells grew into a single layer (about 36 h). For further use, the IECs were grown to 80% confluence in 6-well plates with RPMI-1640 medium (Gibco, USA) containing 10% FBS (Gibco, USA) at 37 °C. Subsequently, the cells were infected with *Neisseria* S1 (MOI = 10), and after 6 h, the cells were incubated with R7I (16 μM) and collected after 12 h. Cells only infected with *Neisseria* were regarded as the positive control, and uninfected cells (without any bacteria/peptide incubation) were taken as the negative control. Total RNA of the cell samples was extracted using Trizol according to the manufacturer’s instructions, followed by reverse transcription using the Superscript Reverse Transcriptase Reagent Kit (Takara, Kyoto, Japan). After that, qRT-PCR was performed with LightCycler 480 SYBR Green I for gene expression (genes related to PPAR pathway and lipid metabolism pathway) by using the Applied Biosystems 7500 system (ABI, San Ramon, CA, USA). The β-actin gene was used as a control gene, and the primers used are listed in Table 1.

To determine whether R7I regulated lipid metabolism through the PPAR signaling pathway, cells were treated with GW9662 (PPARγ inhibitor, MCE, Shanghai, China, 10 μM). After 6 h, cells were infected with *Neisseria* S1 (MOI = 10), and after 6 h, cells were incubated with R7I (16 μM) and collected after 12 h. Expression of lipid metabolism pathway relative genes was detected by qRT-PCR. Furthermore, the lipid droplet of each group was marked by using the Lipid Droplets Green Fluorescence Assay Kit with BODIPY (Beyotime, China) and observed with a fluorescent microscope.

### 2.13. Regulation of Inflammatory Response via PPAR Pathway

To determine whether R7I regulated the inflammatory response through the PPAR signaling pathway, cells in 24-well plates were transfected with the pNF-κB-luc reporter plasmid (0.44 μg/well) and internal reference plasmid pRL-TK (0.06 μg/well) using lipofectamine^®^ LTX & Plus Reagent (Invitrogen, Waltham, MA, USA). At 12 h post transfection, cells were treated with GW9662 for 6 h. Subsequently, cells were infected with *Neisseria* S1 (MOI = 10) for 6 h, followed by incubation with R7I (16 μM), and were collected after 12 h. The firefly luciferase and the Renilla luciferase activities of each group were detected using a Dual-Luciferase^®^ Reporter Assay System (Promega, Madison, WI, USA). Subsequently, the expression of inflammatory cytokines (IL-1β, IL-6, and IL-8) was determined by using ELISA kits according to the manufacturer’s instructions.

Indirect immunofluorescence analysis (IFA) was performed to detect activation of the NF-κB signaling pathway. Cells were collected and washed twice with PBS, and then fixed with 4% paraformaldehyde at room temperature (RT) for 30 min. After that, the cells were treated with 0.2% Triton X-100 for permeabilization at RT for 10 min, followed by blocking with 0.3% bovine serum albumin (BSA) at 37 °C for 1 h. Subsequently, the cells were incubated with primary antibodies (rabbit anti-p65) at RT for 2 h, and then incubated with secondary antibodies (FITC-labeled goat anti-rabbit IgG antibody) (Bioss, Beijing, China) at 37 °C for 1 h. After treatment with DAPI (Beyotime, China) for 15 min and washing with PBS, protein expression and subcellular localization were observed by using a fluorescent microscope.

### 2.14. Statistical Analysis

In this study, the results are shown as the mean ± SD of three replicates per test in a single experiment repeated thrice. Tukey’s multiple comparison tests and one-way analysis of variance (ANOVA) were used to analyze the differences groups. * (*p* < 0.05), ** (*p* < 0.01), and *** (*p* < 0.001) were considered significant and calculated using GraphPad Prism 8.0 software.

## 3. Results

### 3.1. Isolation, Identification, and Biological Characteristics of Neisseria

As mentioned previously, in June 2022, an outbreak of acute diarrheal disease occurred among domestic geese in Heilongjiang Province. Pathological examination showed intestinal swelling in the infected geese, while other tissues did not show significant changes (Figure 1B). RT-PCR analysis ruled out common viral infections, including Avian influenza virus, Newcastle disease virus, Goose parvovirus, Goose paramyxovirus, and Duck plague virus, leading us to speculate that bacterial pathogens caused the disease. Single colonies were isolated via plate streaking and were cultured, followed by identification of morphology and 16S rDNA sequencing. A strain of Gram-negative diplococci was isolated from the intestines, while other tissues were free of the strain (Figure 1C–E). The OD_600_ of the bacterial culture was determined to identify the growth curve of the *Neisseria* S1 strain. The one-step growth curve (Figure 1F) shows that 0–2 h corresponds to the lag phase, 4–12 h to the logarithmic growth phase, and 12–16 h to the plateau phase. Following amplification and sequencing of the 16S rDNA gene of the *Neisseria* S1 strain by RT-PCR, phylogenetic analysis was conducted using MEGA 7 software to explore the evolutionary relationships between the *Neisseria* S1 strain isolated in this study and other *Neisseria* strains available in GenBank. The 16S rDNA gene sequence of *Neisseria* S1 was deposited in GenBank under accession number PP837720. As presented in Figure 1G, *Neisseria* S1 and KH1483 are in the same branch, indicating that the phylogenetic analysis revealed that the 16S rDNA gene of *Neisseria* S1 exhibits the highest sequence homology to the strain KH1483 (which was isolated in *Anser albifrons* in the Arctic in 2015) [22].

The results of the antibiotic susceptibility test are shown in Table 2. The results were judged against Performance Standards for Antimicrobial Susceptibility Testing (M100) [30]. The isolated strain was sensitive to neomycin, erythromycin, azithromycin, cefazolin, doxycycline, fosfomycin, and amikacin but resistant to 10 commonly used antibiotics, including gentamicin, streptomycin, enrofloxacin, amoxicillin, ampicillin, and penicillin. The tolerance of the *Neisseria* S1 strain against antibiotics poses significant challenges in clinical treatment.

### 3.2. Whole-Genome Sequencing Analysis of Neisseria

**Figure 2:**
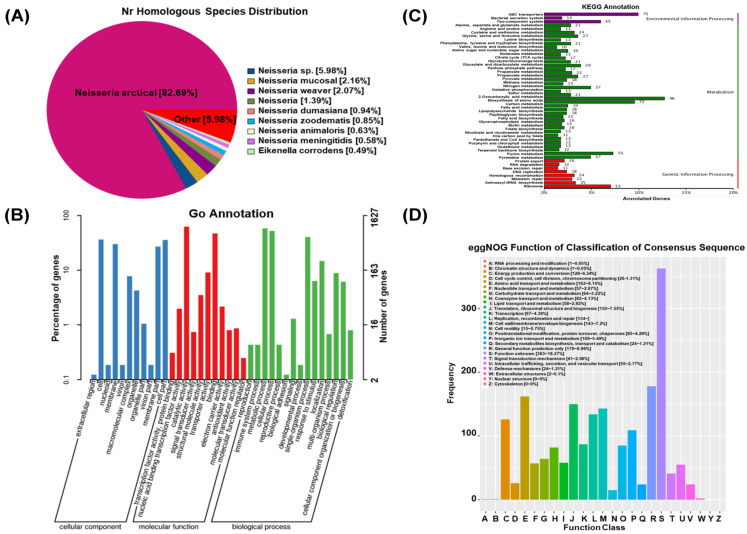
Whole-genome sequences analysis of *Neisseria* S1. (**A**) Nr homologous species distribution. (**B**) GO annotated classification statistics chart. (**C**) Classification of KEGG metabolic pathways. (**D**) Histogram of eggNOG classification statistics.

The complete genome sequence of *Neisseria* S1 was obtained Via next-generation sequencing and deposited in GenBank under accession number PRJNA1153424. The results showed that the total length of *Neisseria* S1 was 2431956 bp, the GC content was 46.8%, and the number of the predicted coding genes was about 2308. Furthermore, there were 44 tandem repeat sequences (total repetitive sequence length is 4330 bp, accounting for 0.18% of the genome), 3 rRNAs (1 5S rRNA, 1 16S rRNA, and 1 23S rRNA) and 50 tRNAs. The annotation results of the Nr database showed that 2224 genes were encoded by *Neisseria* S1, while Nr homologous species distribution (Figure 2A) showed that 82.69% of genes belong to *Neisseria arctica*. The GO database annotated a total of 1627 genes across three classifications: cellular component, molecular function, and biological process (Figure 2B). In the cellular component, they were mainly related to the composition of the cell, membrane, membrane part, cell part, and other factors. In the molecular function, they were mainly related to catalytic activity, binding, transporter activity, and structural molecule activity. In the biological process, they were mainly related to the metabolic process, cellular process, single-organism process, localization, biological regulation, and cellular component organization or biogenesis. As presented in Figure 2C, the KEGG database annotated 1421 genes involved in environmental information processing, metabolism, and genetic information processing. Metabolism is the most enriched item, mainly including the biosynthesis of amino acids, carbon metabolism, and purine metabolism. As presented in Figure 2D, the eggNOG database annotated 1943 genes, with functions predominantly associated with amino acid transport and metabolism, translation, ribosomal structure and biogenesis, cell wall/membrane/envelope biogenesis, and replication, recombination, and repair processes.

### 3.3. Assessment of Anti-Neisseria Activity of R7I In Vitro

Using an improved broth dilution method, the biological activity of peptide R7I against *Neisseria* S1 was evaluated in vitro. The MIC value of peptide R7I against *Neisseria* S1 was 8 μM, and the MBC value was 16 μM (Table 3), indicating that peptide R7I exhibits strong antibacterial activity.

**Figure 3:**
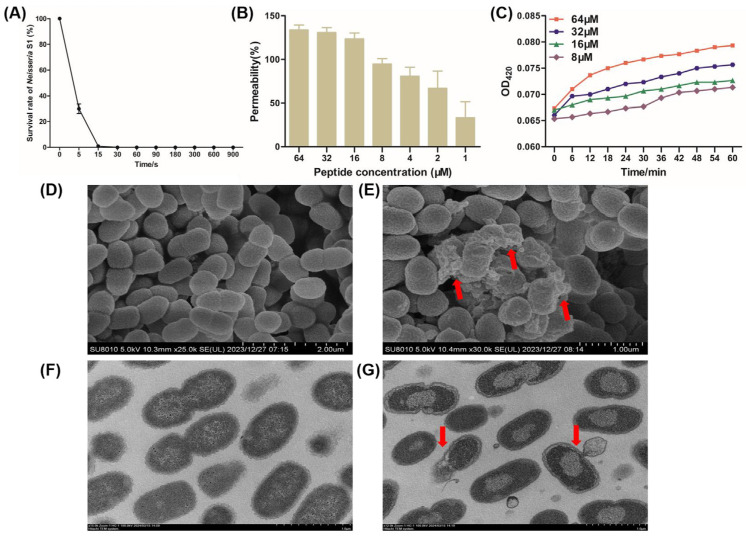
The mechanism of peptide R7I against *Neisseria* in vitro. (**A**) Time-to-kill kinetic curves of the peptide at 1 × MBC against *Neisseria* S1. *Neisseria* S1 was basically completely killed within 15 s, indicating that R7I has extremely high antibacterial efficiency. (**B**) The outer membrane permeability induced by R7I. An NPN uptake assay was used to detect the penetration ability of *Neisseria* S1 in the presence of different concentrations of R7I. The degree of damage to the *Neisseria* outer membrane is positively correlated with the concentration of R7I. (**C**) The inner membrane permeability induced by R7I. An ONPG release assay was used to detect the inner membrane permeability of *Neisseria* S1 in the presence of different concentrations of R7I. R7I induced an increase in inner membrane permeability rapidly at a low concentration. (**D**–**G**) Morphological observation of *Neisseria* S1 by SEM (**D**,**E**) and TEM (**F**,**G**) in the presence of R7I. (**D**,**F**) *Neisseria* S1. (**E**,**G**) *Neisseria* S1 treated with R7I. Red arrows in (**E**,**G**) indicate the release of the contents in the bacteria.

Time-to-kill kinetic curves of peptide R7I at 1 × MBC (16 μM) against *Neisseria* S1 were measured. As shown in Figure 3A, 70% of *Neisseria* S1 cells were sterilized within 5 s and 99% within 15 s, demonstrating the extremely high antibacterial efficiency of R7I. An NPN uptake assay was performed to assess the penetration ability of peptide R71 against *Neisseria* S1. It can be demonstrated that the degree of damage to the *Neisseria* outer membrane is positively correlated with the concentration of antimicrobial peptide R7I. The permeability of the *Neisseria* S1 outer membrane reached over 70% at an R71 concentration of 4 μM (Figure 3B). Furthermore, the inner membrane permeability was measured by the release of ONPG. R7I-induced ONPG was rapidly released at a concentration between 8 and 64 μM, indicating that R7I has the ability to increase the permeability of the inner membrane (Figure 3C). Finally, we used SEM and TEM to observe the effect of peptide R7I on *Neisseria* S1. The surface of *Neisseria* S1 pored and led to the release of intracellular contents due to the effect of peptide R7I (Figure 3E), while the membrane was intact in the control group (Figure 3D). Furthermore, the results of TEM showed that the membrane and cell wall were tightly connected in untreated *Neisseria* S1, and there were no obvious cavities in the cytoplasm (Figure 3F). There was significant cytoplasm–wall separation, cell membrane rupture, and leakage of contents from the ruptured pores in *Neisseria* S1 treated with R7I, which resulted in an uneven distribution of cytoplasm and the formation of a clear blank in the bacterial body (Figure 3G).

### 3.4. Assessment of Anti-Neisseria Activity of R7I In Vivo

**Figure 4:**
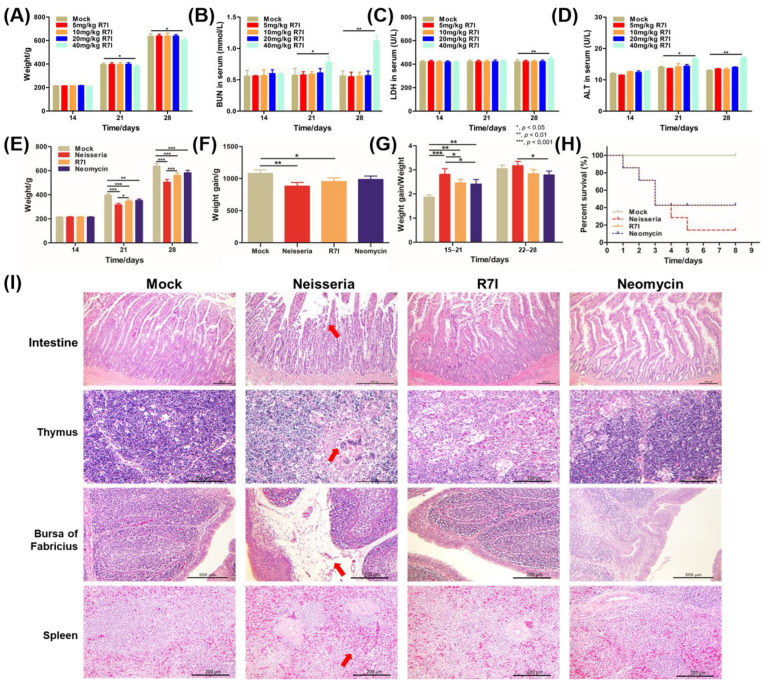
Assessment of anti-*Neisseria* activity of R7I in vivo. (**A**–**D**) Determination of R7I concentration used in vivo. Different doses of R7I were administered orally to geese. Weight changes (**A**) and BUN (**B**), ALT (**C**), and LDH (**D**) levels of geese that received different concentrations of R7I were determined on different days post R7I administration. (**E**–**I**) Protection effect of R7I against *Neisseria* infection. The geese in the Neisseria, R7I, and Neomycin groups were orally infected with 2 × 10^8^ CFU of *Neisseria* S1 for 3 days continuously, while the Mock group were orally immunized with sterile PBS. The R7I and Neomycin groups were orally administered R7I (20 mg/kg) or Neomycin (20 mg/kg), respectively, post *Neisseria* S1 infection. (**E**) Weight changes in geese in each group. (**F**) Weight gain of geese in each group. (**G**) The ratio of weight gain/weight of geese in each group. (**H**) The survival rate of each group. R7I and Neomycin improved the growth performance and survival rates after *Neisseria* infection. (**I**) Histopathological changes in the intestine, thymus, bursas of Fabricius, and spleen of each group post challenge. There were obvious pathological changes in the Neisseria group, slight changes in the Neomycin group, and no obvious changes observed in the Mock group and the R7I group. The red arrow points to typical pathological changes. The scales of intestine, thymus, bursa of Fabricius and spleen were 200 μm, 100 μm, 200 μm and 200 μm, respectively.

As shown in Figure 4A–D, 40 mg/kg of R7I could cause damage to geese and to some extent inhibit their growth. Moreover, 20 mg/kg is the maximum dose that does not have a significant effect on geese, so we chose 20 mg/kg as the final experimental concentration. The anti-*Neisseria* effect of R7I in vivo was determined in a goose model. As shown in Figure 4E–H, compared with the Mock group, the weight, weight gain, and ratio of weight gain/weight of geese decreased in the Neisseria group, while the R7I group and the Neomycin group showed increases, indicating that R7I and Neomycin treatment alleviated the decline in the growth performance of geese caused by *Neisseria* infection. Furthermore, most of the geese infected with *Neisseria* died on the 5th day post infection (Figure 4D). However, the survival rates of geese treated with R7I and Neomycin were about 40% by the 3rd day post infection, suggesting that both R7I and Neomycin can resist the infection of *Neisseria* to a certain extent.

Pathological anatomy showed intestinal swelling in the infected geese, while other tissues did not show significant changes. The results of histopathological observation revealed significant damage in the *Neisseria*-infected group, including villus atrophy and shortening, shedding of mucosal epithelial cells in the intestine, and multiple multinucleated giant cell hyperplasia seen in the thymus medulla. Some follicles of bursa of Fabricius were atrophied, lymphocytes were reduced, lamina propria was obviously edema, and there was a small amount of inflammatory cell infiltration. Additionally, the number of lymphocytes decreased, and red blood cells accumulated in the spleen of the Neisseria group (Figure 4I). Treatment with R7I and Neomycin alleviated the pathological changes caused by *Neisseria* infection. There were no obvious pathological alterations in the R7I group and only slight pathological changes in the thymus and bursa of Fabricius of the Neomycin group. The lymphocytes in bursa of Fabricius and some lymphoid follicles decreased slightly, while there were changes in the thymus cortex and medulla ratio, mild-to-moderate atrophy, and thinning of the cortex. These results indicate that *Neisseria* infection caused extensive damage to the tissues of geese, and both peptide R7I and Neomycin can effectively alleviate the damage caused by *Neisseria* infection.

### 3.5. Regulation of Blood Biochemical Indexes, Antibodies, and Cytokine Levels in Geese

**Figure 5:**
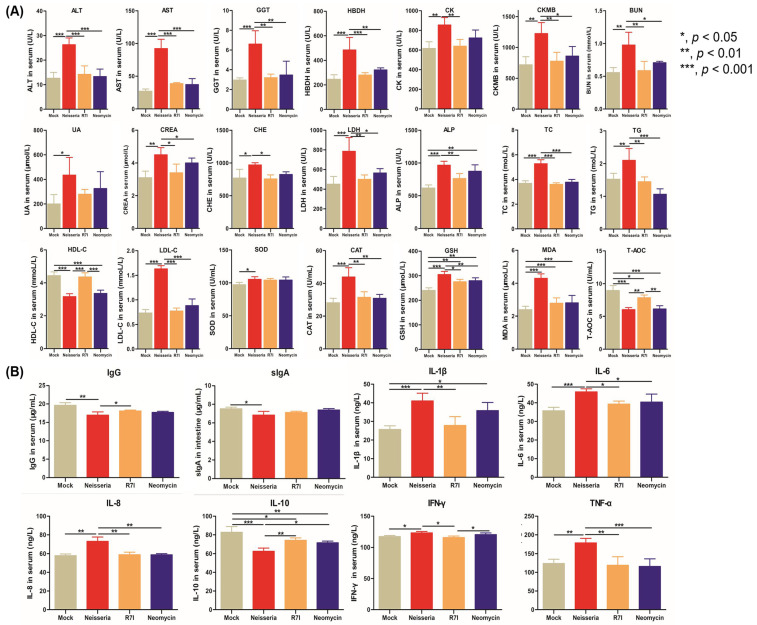
The levels of blood biochemical indexes, antibodies, and cytokines in geese. (**A**) The levels of blood biochemical indexes in geese. R7I and Neomycin alleviated inflammation, the level of lipid metabolism, and the antioxidant capacity and injury of the liver, kidney, and heart caused by *Neisseria* infection. (**B**) The levels of antibodies and cytokines in serum and sIgA in intestinal mucus of immunized geese. R7I and Neomycin regulated the production of antibodies and cytokines in defense against *Neisseria* infection.

The levels of liver-associated indexes (ALT, AST, ALP, GGT, and CHE), kidney-associated indexes (BUN, UA, and CREA), heart-associated indexes (HBDH, CK, and CKMB), and the inflammatory-associated index (LDH) in geese were measured at 7 DPI. The results are presented in Figure 5A. Compared with the Mock group, the levels of these markers increased in the *Neisseria*-infected group; however, treatment with R7I and Neomycin alleviated these changes. Notably, there were no significant differences between the Mock group and the R7I-treated group, indicating that peptide R7I could effectively inhibit inflammation and protect the function of the liver, kidney, and heart in *Neisseria*-infected geese.

The levels of lipid metabolism-associated indexes (TC, TG, HDL-C, and LDL-C) and antioxidant-associated indexes (SOD, CAT, T-AOC, GSH, and MDA) in serum are shown in Figure 5A; compared with the Mock group, the levels of T-AOC and HDL-C were significantly decreased (*p* < 0.001), while other indexes increased in the Neisseria group. R7I and Neomycin alleviated these changes caused by *Neisseria* infection, helping to restore antioxidative balance and correct lipid metabolism disorders.

The levels of IgG antibodies and cytokines (IL-1β, IL-6, IL-8, IL-10, IFN-γ, TNF-α) in serum and sIgA in intestinal mucus were determined. As shown in Figure 5B, compared with the Neisseria group, R7I and Neomycin promoted the production of IgG, indicating that R7I and Neomycin activated humoral immunity in defense against *Neisseria* infection. There were no significant changes in the levels of sIgA in the R7I, Neomycin, and Neisseria groups, indicating that R7I and Neomycin could not induce mucosal immunity in geese. Compared with the Mock group, the levels of pro-inflammatory factors IL-1β, IL-6, IL-8, IFN-γ, and TNF-α were significantly elevated (*p* < 0.05) in the Neisseria group, while the anti-inflammatory factor IL-10 was significantly reduced (*p* < 0.001). Treatment with R7I and Neomycin inhibited the production of pro-inflammatory cytokines and promoted the production of anti-inflammatory cytokines in geese. The increase in Th2-type cellular immune-related cytokines (IL-10) and the decrease in Th1-type cellular immune-related cytokines (IL-8 and IFN-γ) in the R7I group indicate that R7I treatment regulated the cellular immune response to the Th2 subtype in *Neisseria* infection.

### 3.6. R7I Regulates Neisseria-Induced Dysbiosis of the Gut Microbiota

**Figure 6:**
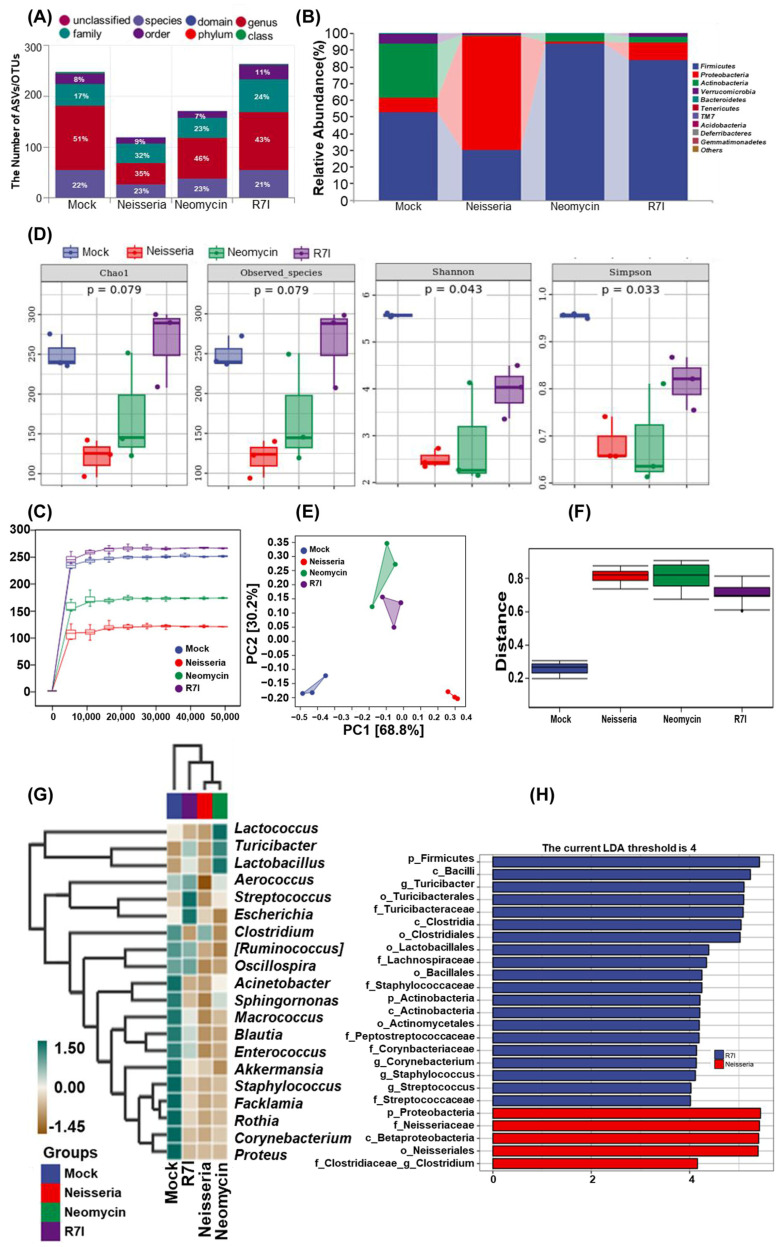
Gut microbiota analysis by 16S rDNA sequencing. (**A**) Statistics on the number of taxonomic units in species composition. (**B**) Taxonomic composition analysis at the phylum level. (**C**) Rarefaction curves of each group. The smoothness of the curve reflects the impact of sequencing depth on the alpha diversity index of the observed samples. The number of ASV/OTU in R7I > Mock > Neomycin and Control at the same sequencing depth. (**D**) The alpha diversity index. Chao1 of the observed species represents richness, while Shannon and Simpson indexes represent diversity. Significance was tested using the Kruskal–Wallis rank-sum test and Dunn’s test as a post hoc test. (**E**) PCA for differences in species abundance composition at the genus level. The closer the projection distance between two points on the coordinate axis, the more similar the species abundance composition between these two samples in the corresponding dimension. (**F**) Differences in species composition analyzed by Permdisp. (**G**) A species composition heatmap. Samples are clustered using UPGMA based on the Euclidean distance of species composition data and arranged according to the clustering results. (**H**) LDA effect size (LEfSe) analysis. The vertical axis represents the classification units with significant differences between groups, while the horizontal axis visually displays the logarithmic scores of LDA analysis for each classification unit in a bar chart. Classification units are sorted according to their score values to describe their specificity in sample grouping. The longer the length, the more significant the difference in the classification unit.

16S rDNA sequencing was used to investigate the effect of R7I treatment on the gut microbiota. The taxonomic annotation unit showed that the Neisseria group reduced the proportion of ASVs at the genus level while increasing them at the family level (Figure 6A). In Figure 6B, the proportion of *Proteobacteria* in the Mock, R7I, and Neomycin groups is shown to be 8.38%, 10.27%, and 1.35%, respectively, while that of Neisseria was 68.47%. *Firmicutes* levels in the R7I (84.03%) and Neomycin (93.55%) groups were significantly higher compared with Neisseria (29.71%) and Mock (52.76%). The ratio of *Firmicutes*/*Bacteroidetes* increased in the R7I and Neomycin groups, compared with the Neisseria and Mock groups. Alpha diversity analysis showed that the diversity index plateaued with increased sequencing depth, ranking from largest to smallest as follows: R7I, Mock, Neomycin, and Neisseria (Figure 6C). Although Chao1 and the observed species index showed no significant differences in the characterization richness between the four groups (Figure 6D), Shannon and Simpson indexes were used to represent microbial diversity, which showed significant differences (*p* < 0.05) in uniformity between the four groups. R7I and Neomycin improved the microbial diversity destroyed by *Neisseria*, with R7I having a more pronounced effect.

As shown in Figure 6E, principal component analysis (PCA) was used to analyze the differences in species abundance at the genus level. The results revealed that the top two principal components accounted for 68.8% and 30.2% of the variation, respectively. Based on the projection on PC1, the distance of sample centers between Neisseria and Mock was greater than that between R7I/Neomycin and Mock, suggesting that the species abundance composition between R7I/Neomycin is more similar to Mock than Neisseria. This indicates that R7I and Neomycin effectively regulated *Neisseria*-induced dysbiosis of the gut microbiota. Permdisp analysis was conducted to analyze the differences in species composition between groups. As depicted in Figure 6F, the difference between Neisseria–Mock (*p* = 0.04) and Neomycin–Mock groups was significant (*p* = 0.04), while R7I–Mock, R7I–Neisseria, and Neomycin–Neisseria did not show significant differences. The Euclidean distances were clustered by UPGMA, and then species composition analyses were performed. The top 20 genera in terms of relative abundance at the genus level for the four groups are shown in Figure 6G. All classification levels were analyzed simultaneously by LDA effect size (LEfSe). As can be found in Figure 6H, while the LAD score was >4, the difference advantage of the Neisseria group was *Proteobacteria*, *Betaproteobacteria*, *Neisseriaceae*, and *Neisseriales*, while the difference advantage of the R7I group was *Firmicutes*, *Bacilli*, *Turicibacter*, *Clostridia*, *Lactobacillales*, and *Staphylococcaceae*.

### 3.7. R7I Regulates Transcriptome Changes Induced by Neisseria

**Figure 7:**
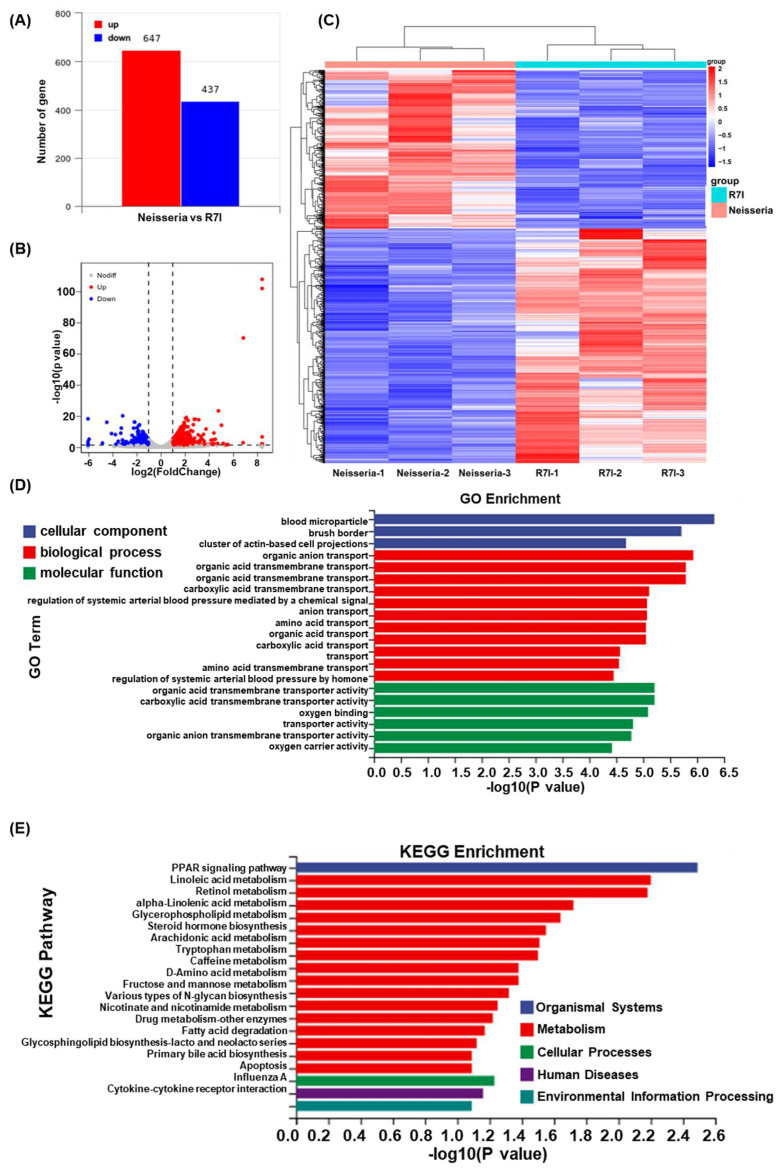
Transcriptome analysis of intestines of the Neisseria and R7I groups. (**A**) Statistics for the DEGs according to the RNA-seq-based transcriptomics data for the R7I-treated group compared with the Neisseria group. The red histogram represents the number of upregulated DEGs, while the blue histogram represents the number of downregulated DEGs. (**B**) A volcano plot of the DEGs. The red dots represent significantly upregulated genes, the blue dots represent significantly downregulated genes, and the gray dots represent insignificant DEGs. (**C**) A cluster heatmap of differential genes in the transcriptome of the Neisseria and R7I groups. GO enrichment (**D**) and KEGG pathway enrichment (**E**) analysis of the DEGs in the RNA-seq transcriptomics data of the R7I-treated group compared with the Neisseria group.

Transcriptome sequencing of goose intestines from the Neisseria and R7I groups was performed using the Illumina NovaSeq platform. As presented in Figure 7A, a total of 1084 DEGs were obtained in the *Neisseria*-infected geese, with 647 genes significantly upregulated and 437 genes downregulated. The distribution of DEGs is visualized in the volcano plot (Figure 7B), while the clustering of these DEGs is presented in Figure 7C. GO annotation and KEGG pathway enrichment analyses were performed to analyze the biological functions of the DEGs. As presented in Figure 7D, GO terms associated with the DEGs were primarily related to blood microparticles, brush borders, organic anion transport, and carboxylic acid transmembrane transport processes. Additionally, the KEGG pathway enrichment analysis (Figure 7E) revealed that the DEGs were primarily associated with the PPAR signaling pathway, linoleic acid metabolism, retinol metabolism, alpha-linolenic acid metabolism, apoptosis, and cytokine–cytokine receptor interaction pathways.

### 3.8. R7I Regulates Lipid Metabolism via PPAR Signaling Pathway

**Figure 8:**
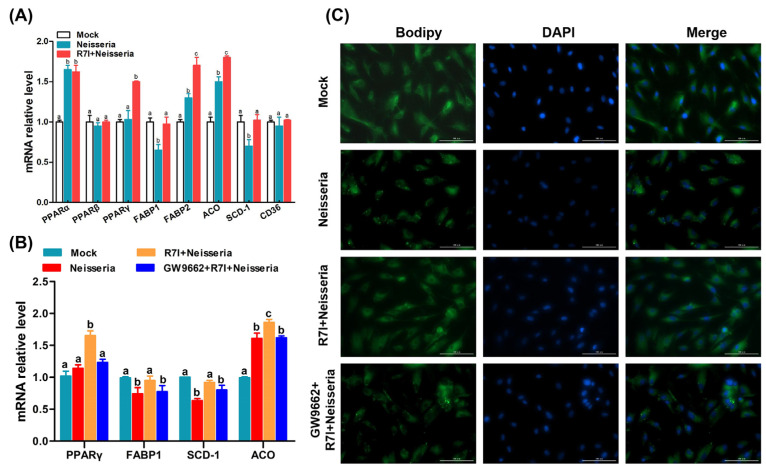
Regulation of lipid metabolism and PPAR signaling pathway. (**A**) Regulation of transcription changes in PPAR signaling pathway and lipid metabolism-related genes induced by R7I. (**B**) Relationship between PPARγ activity and lipid metabolism. (**C**) Distribution of lipid droplets in IECs by BODIPY staining. Different letters (a, b, c) indicate significant differences (*p* < 0.05) between different groups. The scale bar is 100 μm.

Based on the results of transcriptome sequencing, we further determined the regulation of PPAR pathway and lipid metabolism changes in R7I-treated IECs. As shown in Figure 8A, compared with the Neisseria group, the transcription of PPAR pathway-related genes (in particular the PPARγ genes) and lipid metabolism-related genes was changed by R7I. We further evaluated the function of PPARγ in *Neisseria* infection and R7I treatment. Figure 8B shows that R7I significantly upregulated the expression of PPARγ, and the effect was inhibited by treatment with GW9662 (PPARγ inhibitor). Therefore, R7I enhanced the expression of FABP1 and ACO and inhibited the expression of SCD-1, indicating that R7I regulates the excessive oxidation, production, and transport of fatty acids caused by *Neisseria* infection, as well as lipid metabolism disorder. Treatment with the PPARγ inhibitor inhibited the protective effect exerted by R7I, indicating that R7I regulates lipid metabolism disorder caused by *Neisseria* through the PPAR pathway. In addition, the results of lipid droplet staining directly showed that R7I regulated the disorder of lipid droplet expression caused by *Neisseria* infection, and the treatment with the PPARγ inhibitor inhibited the regulation of R7I (Figure 8C).

### 3.9. R7I Regulates Inflammatory Response via PPAR Signaling Pathway

**Figure 9:**
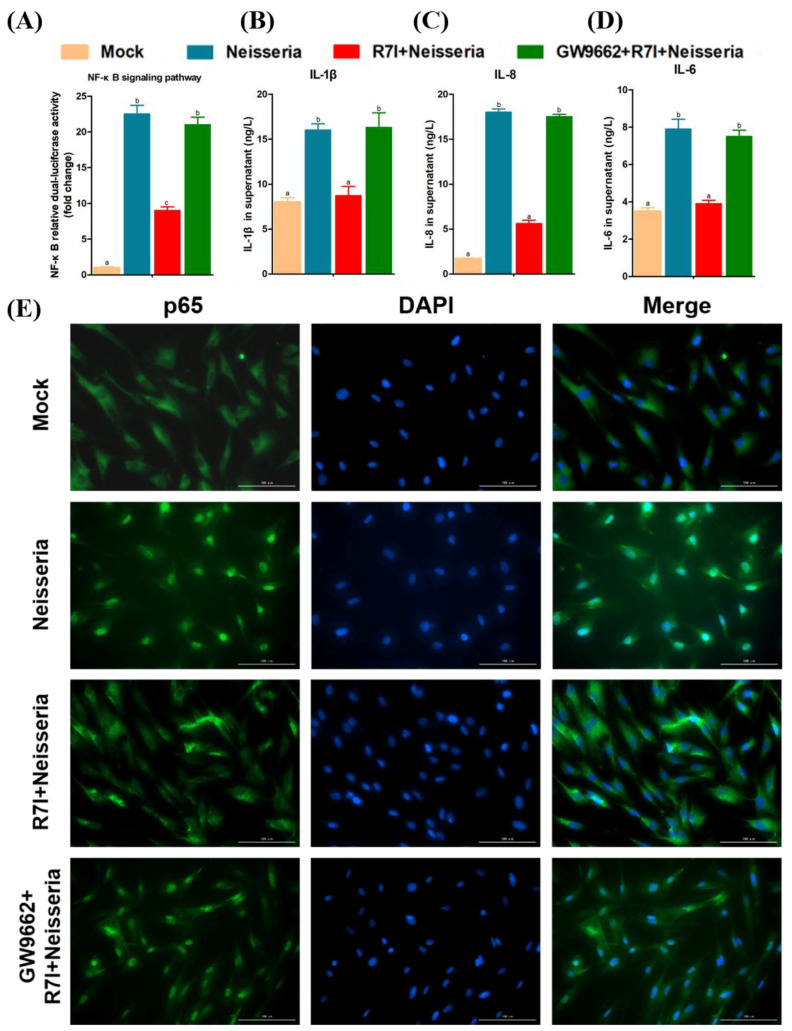
Regulation of NF-κB signaling pathway by R7I Via activation of PPARγ. (**A**) NF-κB relative dual-luciferase activity in geese IECs. (**B**–**D**) Release of inflammatory cytokines (IL-1β, IL-8, and IL-6) in geese IECs. (**E**) Detection of p65 nuclear translocation by IFA. Different letters (a, b, c) indicate significant differences (*p* < 0.05) between different groups. The scale bar is 100 μm.

Besides lipid metabolism, PPARγ also could inhibit the NF-κB signaling pathway to alleviate the inflammatory response. Therefore, we further determined the effect of PPARγ on the NF-κB pathway and inflammatory factor expression. R7I downregulated the activation of NF-κB and the expression of inflammatory cytokines (IL-1β, IL-6, and IL-8), and the PPARγ inhibitor blocked this mitigation effect (Figure 9A–D). The results of the IFA (Figure 9E) showed that R7I prevented the transmission of p65 from cytoplasm to the nucleus, indicating the inhibition of NF-κB pathway. The use of the PPARγ inhibitor alleviated the prevention of NF-κB pathway signaling by R7I.

## 4. Discussion

The overuse of antibiotics is one of reasons for the emergence of antibiotic-resistant bacteria, followed by the lack of available drugs used in clinical treatment. It is crucial to identify alternatives for antibiotics. AMPs are widely considered green and broad-spectrum natural antibiotics, but face challenges like poor proteolytic resistance in vitro, high toxicity and inactivation in vivo, and difficulties in oral administration. AMP R7I designed in our lab overcomes the shortcomings of traditional AMPs, has been confirmed to inhibit the proliferation of intestinal pathogens Via oral treatment in mice, and has become a new oral antimicrobial agent for treating bacterial infection [1,2,6].

*Neisseria* is a multi-host–pathogen that causes widespread infection worldwide [14,15,16,17,18,19,20,21,22]. An outbreak of an acute diarrheal disease among domestic geese occurred in Northeast China in 2022. The causative agent of this epidemic outbreak was identified as a *Neisseria* strain named *Neisseria* S1, and this is the first report of pathogenic *Neisseria* infection in a captive goose (*Anser cygnoides orientalis*). *Neisseria* S1 shares the highest genetic similarity with the strain KH148 (which was isolated in *Anser albifrons* in the Arctic in 2015) [22]. Based on the geographical characteristics of Heilongjiang Province, we suspect that the *Neisseria* S1 was spread by wild geese (*Anser albifrons*) from other cities or countries. In the present study, we characterized *Neisseria* S1. The results show that *Neisseria* S1 is Gram-negative diplococcus, not sensitive to 10 commonly used antibiotics such as enrofloxacin, gentamicin, and penicillin. The emergence of multiple-antibiotic-resistant *Neisseria* poses a challenge to clinical treatment.

AMPs are a potential drug for the treatment of *Neisseria* infection. However, most of the currently available peptides are natural products, and *Neisseria* has the ability to resist the effect of natural AMPs (such as protegrins, a peptide found in porcine leukocytes; Cyclic BmKn-2, a peptide designed based on BmKn-2 which was isolated from *Buthus martensii* Kasch) in vitro [27,32]. There is still a lack of research on the development of artificially designed peptides against *Neisseria* and/or other Gram-negative cocci infections. Therefore, we chose *Neisseria* S1 (a wild strain) as the model bacteria to identify whether artificially designed peptides can be used for the treatment of Gram-negative cocci infection in a clinical setting.

In this study, we evaluated the anti-*Neisseria* ability of peptide R7I in vivo and in vitro. Peptide R7I has a significant ability to sterilize 99% of *Neisseria* within 15 s at a concentration of 16 μM, indicating its strong antibacterial activity and high antibacterial efficiency. It destroyed the cell membranes, eventually causing bacterial death. This is also one of the reasons why antimicrobial peptides are less likely to induce bacterial resistance compared with antibiotics [33]. Research shows that antimicrobial peptides not only kill pathogens in vivo but also improve the growth performance of the host by improving intestinal tissue structure and regulating intestinal microbiota, among others [1]. Feeding with R7I inhibited the pathological changes in tissues (intestine, thymus, bursa of Fabricius, and spleen), regulated growth performance, and increased the survival rates in geese. In addition, peptide R7I has been shown to have significant therapeutic effects in *E. coli* or *Salmonella typhimurium*-infected mice [1,2]. These results provide a basis for the practical application of peptide R7I in vivo.

Additionally, intestinal pathogens are capable of causing damage to other tissues and initiating metabolic disorders. The increase in AST, ALT, ALP, GGT, and CHE in serum indicated liver damage due to *Neisseria* infection [34,35,36,37]. R7I and Neomycin alleviated liver damage from *Neisseria* infection. R7I also significantly reduced BUN and CREA but did not affect UA, which reflects changes in renal function [38,39]. R7I was found to slow down the rise in HBDH, CK, and CKMB levels, suggested that R7I could protect cardiac function against damage by *Neisseria* infection [40,41]. In addition, R7I and Neomycin reduced the increase in LDH, indicating that R7I and Neomycin inhibited the inflammatory response induced by *Neisseria* infection [42].

Abnormal lipid metabolism often manifests as an accumulation of TC, TG, and LDL-C and a decrease in HDL-C in serum [43]. R7I significantly alleviated abnormal lipid metabolism caused by *Neisseria* infection. *Neisseria* infection resulted in a decrease in T-AOC and an increase in R7I and Neomycin, indicating that R7I and Neomycin improve total antioxidant capacity [44]. GSH, SOD, and CAT are important enzymes in the antioxidant defense system. MDA is the key end product of lipid peroxidation and a biomarker for oxidative stress [45]. CAT and GSH levels in the Neisseria group were significantly higher than in the other three groups. R7I and Neomycin partially improved the damage resulting from oxidative stress abnormalities caused by *Neisseria*. However, no significant improvement was observed for SOD levels. *Neisseria* infection may induce ROS production and reduce antioxidant enzyme activity, thereby leading to lipid oxidation and MDA accumulation [45]. There were no significant differences in MDA levels among R7I, Neomycin, and Mock groups. Overall, R7I and Neomycin had a positive regulatory effect on oxidative stress caused by *Neisseria* infection. Antibodies and cytokines play an important role in the body’s immune defense against infection [46,47]. In this study, the levels of IgG, IL-1β, IL-6, IL-8, IL-10, IFN-γ, and TNF-α in serum and sIgA in intestinal mucus were determined. R7I induced humoral immunity and Th2-type cellular immunity in defense against *Neisseria* infection.

Pathogenic microorganisms can cause dysbiosis of the gut microbiota. Bacterial dysbiosis in inflammatory bowel disease is characterized by a reduction in bacterial diversity, a decline in the *Firmicutes* phylum, and an increase in *Proteobacteria* phylum [48,49]. In this study, compared with the Mock group, the gut microbiota of the Neisseria group also exhibited this characteristic. R7I and Neomycin inhibited the changes in the composition and abundance of the intestinal flora caused by *Neisseria* infection. Specifically, the administration of R7I and Neomycin decreased the percentage of *Proteobacteria* and increased the percentage of *Firmicutes*. Further, the results of the taxonomic annotation unit of the species, the rarefaction curve, Chao1, and the total number of ASVs demonstrated the protective effect of R7I. *Turicibacter* is related to glucose and lipid metabolism and can produce short-chain fatty acids, while *Lactobacillus* has been shown to have positive effects on various diseases, including aiding digestion, inhibiting cancer, regulating gut microbiota, and alleviating secondary diseases [50,51,52]. In addition, probiotics such as *Lactobacillus* have been shown to have certain adjuvant therapeutic effects on IBD by protecting the intestinal mucosal barrier and inhibiting inflammatory signals [53]. R7I significantly reduced the abundance of *Neisseria* and increased the abundance of beneficial bacteria in the gut, such as *Lactobacillus* and *Turicibacter*, and improved the composition and regulated the balance of the gut microbiota. The results of the gut microbiota analysis demonstrated that *Neisseria* disrupted the diversity, abundance, and composition of gut microbiota in geese. Treatment with peptide R7I effectively alleviated this disruption and outperformed Neomycin in restoring microbial diversity and abundance, which is more conducive to maintaining intestinal homeostasis.

A total of 1084 DEGs obtained by RNA-Seq were mainly enriched in the PPAR signaling pathway, lipid metabolism, apoptosis, and other pathways. The results of qRT-PCR showed that R7I upregulated the expression of the PPARγ gene and regulated lipid metabolism disorder. As is known, PPARγ could regulate lipid metabolism in various infections [54]. We further determined the function of PPARγ which was upregulated by R7I during lipid metabolism disorder induced by *Neisseria* infection. R7I enhanced the transcription of FABP1 and ACO and inhibited the level of SCD-1, while the effect was inhibited by treatment with GW9662 (PPARγ inhibitor), indicating that R7I regulated fatty acid (FA) transport [55] and the lipolytic [56] and FA synthesis [57] disorder caused by *Neisseria* infection Via PPARγ; it then regulated lipid metabolism disorder and the distribution of lipid droplets. Beyond lipid metabolism, PPARγ also regulates the expression of inflammatory cytokines through the NF-κB signaling pathway [58]. We further determined the effect of PPARγ on the NF-κB pathway and inflammatory factor activation. R7I significantly inhibited *Neisseria*-induced overexpression of the pro-inflammatory cytokines IL-1β, IL-6, and IL-8 by modulating the PPARγ/NF-κB pathway. Notably, this effect was decreased when cells were treated with the PPARγ inhibitor GW9662. However, due to the limitations of goose-derived antibodies, we were unable to perform protein expression detection, and this will be one of the tasks we need to undertake in the future.

## 5. Conclusions

In conclusion, this study discovers a new pathogenic *Neisseria* strain from goose intestines and demonstrates that the goose-derived antibiotic-resistant *Neisseria* can be inhibited by antimicrobial peptide R7I, both in vivo and in vitro. Oral treatment of R7I can alleviate *Neisseria* infection by improving growth performance, regulating immune responses and metabolic functions, and improving intestinal homeostasis. The comprehensive therapeutic effect of peptide R7I was comparable with, if not superior to, that of Neomycin at the same dose. R7I presents potential application prospects in treating multi-antibiotic-resistant bacterial infections. In addition, R7I regulated *Neisseria*-induced lipid metabolism disorder and inflammatory responses through activation of the PPARγ protein. These results provide a basis for using R7I as an alternative antibiotic in clinical practice.

## Figures and Tables

**Table 2:** Antibacterial circle diameter of antibiotics against *Neisseria* S1.

Types of Antibacterial Drugs	Antibiotics	Diameter of Inhibition Zone (mm)	Judgment Standard [30]			Sensitivity
			Resistance (R)	Intermediate (I)	Sensitive (S)	
Aminoglycosides	Gentamicin	0.27 ± 0.18	≤12	13–14	≥15	R
	Neomycin	18.26 ± 0.33	≤12	13–16	≥17	S
	Amikacin	18.37 ± 0.25	≤14	15–16	≥17	S
	Streptomycin	0.12 ± 0.02	≤11	12–14	≥15	R
Quinolones	Ofloxacin	13.46 ± 0.19	≤12	13–15	≥16	I
	Ciprofloxacin	9.58 ± 0.24	≤15	16–20	≥21	R
	Enrofloxacin	5.36 ± 0.75	≤27	28–36	≥37	R
β-Lactams	Amoxicillin	12.07 ± 0.42	≤18	19–25	≥26	R
	Ampicillin	6.83 ± 0.69	≤18	19–25	≥26	R
	Cefazolin	30.43 ± 0.87	≤14	15–17	≥18	S
	Penicillin	0.68 ± 0.17	≤26	27–46	≥47	R
Macrolides	Erythromycin	27.55 ± 0.41	≤13	14–22	≥23	S
	Azithromycin	28.21 ± 0.68	≤13	14–17	≥18	S
Sulfonamides	Compound Sulfamethoxazole Tablets	0.52 ± 0.11	≤23	24–32	≥33	R
Tetracyclines	Doxycycline	17.62 ± 0.44	≤12	13–15	≥16	S
Polyphosphate	Fosfomycin	21.98 ± 0.19	≤12	13–15	≥16	S
Glycopeptides	Vancomycin	9.75 ± 0.53	≤14	15–16	≥17	R
Lincomycin	Lincomycin	9.82 ± 0.16	≤23	24–30	≥31	R

**Table 3:** Determination of MIC and MBC.

	R7I (μM)								
	64	32	16	8	4	2	1	TSB with Bacteria	TSB Without Bacteria
OD_600_ for MIC	0.1721 ± 0.0104	0.1683 ± 0.0082	0.1651 ± 0.0037	0.1642 ± 0.0053	0.3294 ± 0.0041	0.4422 ± 0.0059	0.5598 ± 0.0115	0.6927 ± 0.0252	0.1505 ± 0.0018
OD_600_ for MBC	0.1898 ± 0.0115	0.1883 ± 0.0056	0.1878 ± 0.0107	0.4381 ± 0.0096	0.6843 ± 0.0059	0.9958 ± 0.0126	1.6251 ± 0.1094	2.5837 ± 0.2069	0.1882 ± 0.0962

## Data Availability

The data that support the findings of this study are available from the corresponding author upon reasonable request. The datasets presented in this study can be found in online repositories. The names of the repository/repositories and accession number(s) can be found at https://www.ncbi.nlm.nih.gov (accession ID: PRJNA1118495, PRJNA1118908, and PRJNA1153424).

## Abbreviations

The following abbreviations are used in this manuscript:

ALP	Alkaline phosphatase
ALT	Alanine aminotransferase
AMPs	Antimicrobial peptides
AST	Aspartate aminotransferase
BUN	Urea nitrogen
CAT	Catalase
CHE	Cholinesterase
CK	Creatine kinase
CKMB	Creatine phosphokinase isoenzyme
eggNOG	Evolutionary Genealogy of Genes: Non-supervised Orthologous Groups
ELISA	Enzyme-linked immunosorbent assay
GGT	Glutamyltransferase
GO	Gene ontology
CREA	Creatinine
GSH	Glutathione
HBDH	α-hydroxybutyrate dehydrogenase
HE	Hematoxylin-eosin
HDL-C	High-density lipoprotein cholesterol
HPLC	High-performance liquid chromatography
IECs	Intestinal epithelial cells
KEGG	Kyoto Encyclopedia of Genes and Genomes
LEfSe	LDA effect size
LDH	Lactate dehydrogenase
LDL-C	Low-density cholesterol
MBC	Minimum bactericidal concentration
MDA	Malondialdehyde
MIC	Minimum inhibitory concentration
NPN	1-N-phenylnaphthylamine
Nr	Non-redundant
ONPG	o-nitrophenyl-β-Dgalactoside
PAMPs	Pathogen associated molecular patterns
PBMCs	Peripheral blood monocytes
PCA	Principal component analysis
RES	Resveratrol
ROS	Reactive oxygen species
SEM	Scanning electron microscope
SOD	Superoxide dismutase
T-AOC	Total antioxidant capacity
TC	Cholesterol
TEM	Transmission electron microscopy
TG	Triglyceride
TSB	Tryptic Soy Broth
UA	Uric acid
